# Sternoclavicular joint septic arthritis following paraspinal muscle abscess and septic lumbar spondylodiscitis with epidural abscess in a patient with diabetes: a case report

**DOI:** 10.1186/1471-227X-12-7

**Published:** 2012-06-15

**Authors:** Nobuki Shioya, Yoriko Ishibe, Shigenori Kan, Takayuki Masuda, Naoya Matsumoto, Gaku Takahashi, Hideyuki Makabe, Yasuhiko Yamada, Shigeatsu Endo

**Affiliations:** 1 Department of Critical Care and Emergency, Iwate Prefectural Advanced Critical Care and Emergency Center, Iwate Medical University, 19-1 Uchimaru, Morioka, 020-8505, Japan

**Keywords:** Sternoclavicular joint, Septic arthritis, Spondylitis, Epidural abscess, Epidural anesthesia, Staphylococcus aureus

## Abstract

**Background:**

Septic arthritis of the sternoclavicular joint (SCJ) is extremely rare, and usually appears to result from hematogenous spread. Predisposing factors include immunocompromising diseases such as diabetes.

**Case presentation:**

A 61-year-old man with poorly controlled diabetes mellitus presented to our emergency department with low back pain, high fever, and a painful mass over his left SCJ. He had received two epidural blocks over the past 2 weeks for severe back and leg pain secondary to lumbar disc herniation. He did not complain of weakness or sensory changes of his lower limbs, and his bladder and bowel function were normal. He had no history of shoulder injection, subclavian vein catheterization, intravenous drug abuse, or focal infection including tooth decay. CT showed an abscess of the left SCJ, with extension into the mediastinum and sternocleidomastoid muscle, and left paraspinal muscle swelling at the level of L2. MRI showed spondylodiscitis of L3-L4 with a contiguous extradural abscess. *Staphylococcus aureus* was isolated from cultures of aspirated pus from his SCJ, and from his urine and blood. The SCJ abscess was incised and drained, and appropriate intravenous antibiotic therapy was administered. Two weeks after admission, the purulent discharge from the left SCJ had completely stopped, and the wound showed improvement. He was transferred to another ward for treatment of the ongoing back pain.

**Conclusion:**

Diabetic patients with *S. aureus* bacteremia may be at risk of severe musculoskeletal infections via hematogenous spread.

## Background

Septic arthritis of the sternoclavicular joint (SCJ) is an unusual infection [1-3] which is not well understood, but appears to result from either hematogenous or contiguous spread [4]. Spondylodiscitis usually results from hematogenous spread of infections of the skin and subcutaneous tissues [5]. Common predisposing factors include immunocompromising diseases such as diabetes [6,7]. *Staphylococcus aureus* is the most common pathogen in septic spondylitis and septic arthritis, and the most common portal of entry is through the skin.

## Case presentation

A 61-year-old Japanese man was transported to our critical care and emergency center by ambulance with fever, exacerbation of pain in his lower back and both legs, and a painful mass over his left SCJ.

Approximately 3 months previously, he had consulted an orthopedic surgeon because of low back and leg pain. He had been diagnosed with disc herniation at L4-L5, and had been hospitalized for bed-rest and treatment. While hospitalized, he had received several intravenous injections of sodium salicylate, but no peripheral intravenous catheter had been inserted. About 2 months after discharge, he had been referred to our outpatient anesthesiology department because of ongoing leg pain. Two weeks before presentation, he had received his first epidural block using 6 ml of 0.8% mepivacaine hydrochloride at the L4-L5 level, injected via the paramedian approach. Two days before presentation, a second epidural block using 5 ml of 0.8% mepivacaine hydrochloride had been administered at the same level. The skin had been disinfected with 5% chlorhexidine solution prior to needle insertion on both occasions. His medical history included hypertension and diabetes, for which he was taking antihypertensive and hypoglycemic agents, respectively. He had no history of trauma, shoulder injection, subclavian vein catheterization, or intravenous drug abuse. He did not have any focal dental infection or signs of tooth decay. He was unable to walk, due to increased leg pain. The day prior to admission, he experienced a fever of 39 °C.

On examination, he was in moderate respiratory distress and mildly diaphoretic, with a blood pressure of 97/51 mmHg, pulse rate of 95 beats/min, respiratory rate of 28 breaths/min, temperature of 39.5 °C, and oxygen saturation of 80% on room air. His oxygen saturation improved to 92% with oxygen administration (2 l/min by nasal cannula). Examination of the oral cavity and pharynx was normal, and there was no cervical lymphadenopathy. Chest examination was unremarkable except for swelling and severe tenderness over the left SCJ. Lumbar spine examination showed stiffness, with tenderness over the vertebrae. Movement of the lower back and pressure over the lumbar spine caused pain. The straight leg raising test and femoral nerve stretch test were inconclusive bilaterally because of lower back muscle spasm. His lower limb muscle power, knee and ankle reflexes, and sensation were normal. Bladder and bowel function were normal.

**Figure 1:**
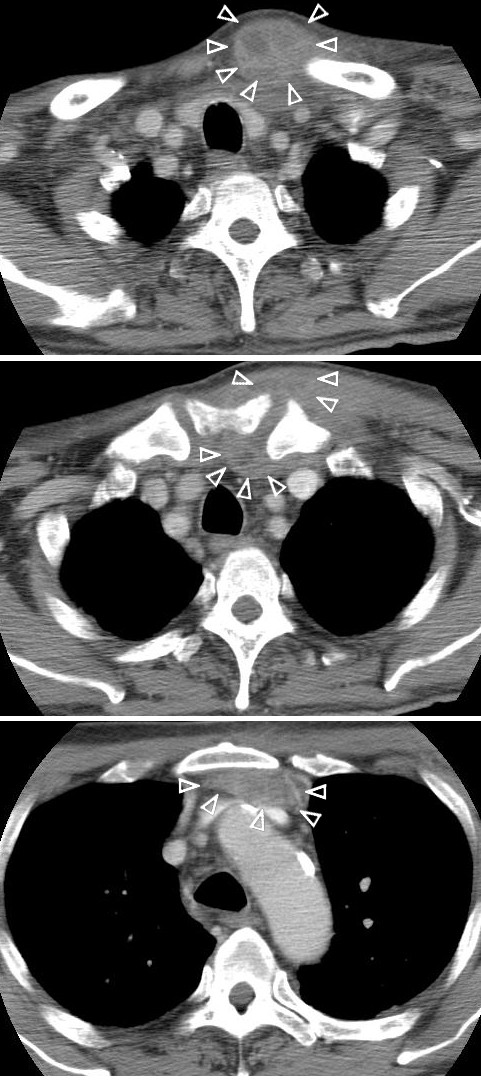
**Thoracic CT scan: A CT scan using intravenous contrast shows an abscess ( *allow head: △* ) around the left SCJ (A and B)**. **The abscess is compartmental structure.** The rim of the mass is slightly enhanced, but the center of the abscess is not enhanced. The abscess extends to the left sternocleidomastoid muscle (**A**) and retrosternal region (**C**).

**Figure 2:**
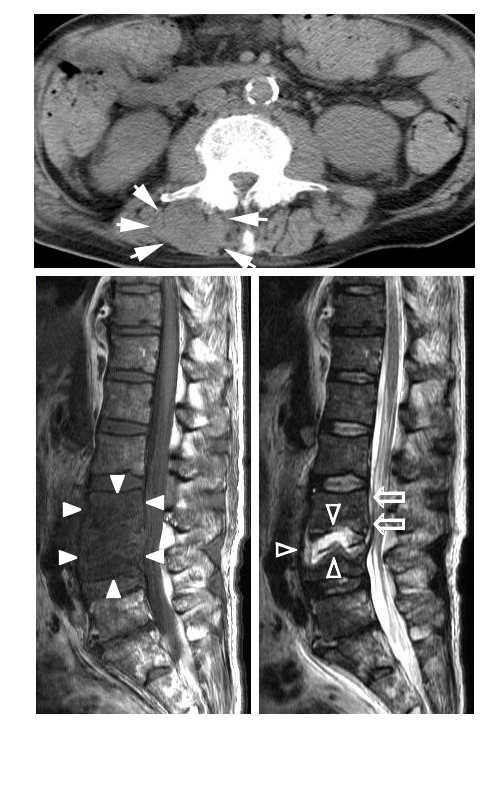
**Lumbar CT scan and MRI: CT demonstrates swelling ( *white allow:* ↑) of the left paraspinal muscle around the L2 level**. (**A**) MRI reveals spondylitis (*white allow head:* ▴) lesions involving the L3-L4 vertebrae (**B**) with ventral contiguous epidural (*framed allow: ⇑*) and disc (*framed allow head: △*) involvement (**C**).

Laboratory testing showed the following results: plasma white blood cell count (WBC) 18,490/mm^3^, platelet count 541,000/mm^3^, hemoglobin 9.0 g/dl, C-reactive protein 22.9 mg/dl, fasting blood glucose 335 mg/dl, glycosylated hemoglobin 8.1%, blood urea nitrogen 23.7 mg/dl, creatinine 0.73 mg/dl, glutamic-oxaloacetic transaminase 59 IU/l, glutamic-pyruvic transaminase 62 IU/l, cholinesterase 134 IU/l, alkaline phosphatase 600 IU/l, lactate dehydrogenase 381 IU/l, and creatine kinase 566 IU/l. US examination of the left SCJ suggested pyogenic arthritis with involvement of the sternocleidomastoid muscle. The chest X-ray was normal, and there were no abnormalities on ECG or cardiac US. CT showed erosion and abscess formation of the SCJ with extension of the abscess into the mediastinum (Figures 1A and 1B) and sternocleidomastoid muscle (Figure 1C). Abdominal CT showed swelling of the left paraspinal muscle at L1-L3 (Figure 2A). MRI showed spondylitis of the L3-L4 vertebrae (Figure 2B) with a focal epidural collection and L3-L4 discitis (Figure 2C).

**Figure 3:**
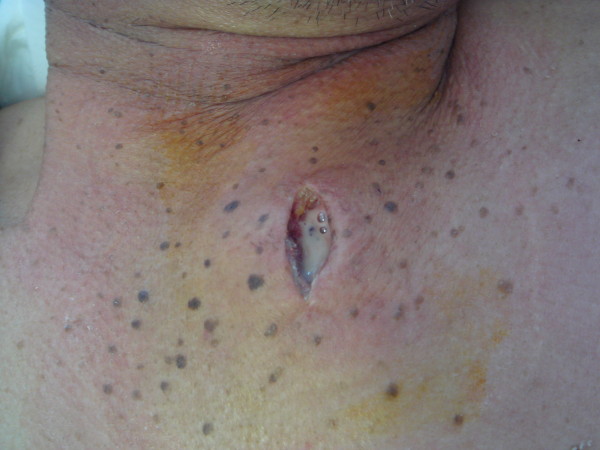
**The left SCJ.** Aspiration of the SCJ and culture of the obtained fluid yielded a growth of *S. aureus.*

Aspiration of the SCJ revealed a turbid fluid (approximately 3 ml) (Figure 3) with clusters of gram-positive cocci on microscopy. The SCJ was incised and drained, and the abscess cavity was enlarged to include the abscess of the left sternocleidomastoid muscle. Sulbactam/ampicillin administration was started immediately. Aspirated pus, urine, and two sets of blood cultures all indicated *S. aureus* infection. According to the results of antibiotic susceptibility testing, the patient’s antibiotic therapy was changed to cefotiam. After one week of antibiotic therapy, blood and wound cultures were negative for pathogens. Follow-up MRI clearly showed abscess formation in the left paraspinal muscle at L1-L3, but culture of fluid aspirated from the abscess showed no growth.

The patient’s general condition improved significantly and his fever subsided after 4 days. The wound at the SCJ was irrigated daily. Twelve days after the onset of treatment, his plasma WBC was 7,050/mm^3^ with 78% neutrophils. Two weeks after admission, there was a still a non-tender swelling over the left SCJ, but the purulent secretion had completely resolved. Neurological examination of the lower limbs was unchanged. Eight weeks after admission, he was transferred to the spinal surgery unit to undergo evaluation for operative treatment of his spondylitis and epidural abscess.

## Discussion

It is likely that this patient’s epidural block caused the paraspinal muscle abscess and nearby lumbar spondylodiscitis, and that this iatrogenic infection spread hematogenously to the SCJ.

Septic arthritis most commonly affects the weight-bearing joints of the lower limb, which account for 61-79% of all reported cases of septic arthritis [8]. The knee is the most commonly affected joint, followed by the hip, shoulder, wrist, ankle, and elbow. There is usually no limiting basement plate under the well-vascularized synovial membrane, facilitating the entry of hematogenously carried bacteria into the joint space [9].

Septic arthritis of the SCJ is extremely rare, comprising 0.5–1% of all joint infections [10], but results in abscess formation in 20% of cases [11,12]. The SCJ is the only joint connecting the trunk with the pectoral girdle, and is therefore involved in all major movements of the upper limb. The function of the articular disc on the clavicular side of the SCJ is to resist the compressive load [13]. SCJ infection can cause life-threatening complications, because the joint capsule is unable to distend and infection spreads beyond the joint quickly, leading to fistula formation, cutaneous abscess or, rarely, mediastinitis and superior vena cava syndrome [14,15].

The pathogenesis of SCJ infection is not well understood, but it appears to result from either hematogenous or contiguous spread. Various factors have been identified as predisposing to the development of SCJ infections, including immunocompromising diseases such as diabetes, rheumatoid arthritis, renal dysfunction, and human immunodeficiency virus infection [6].The SCJ can be seeded with microorganisms via the subclavian vein following injection into the veins of the upper extremity or neck (including intravenous drug abuse), clavicular fractures, subclavian vein catheterization, or scratches or animal bites to the hand or arm [6,16]. SCJ infection is generally unilateral, affecting the right side in approximately 60% of cases. This difference in occurrence between sides is less apparent in intravenous drug abusers [4,17]. According to El Ibrahimi, bacteremia was the most commonly assumed mechanism of infection [17]. As in most joints, septic arthritis of the SCJ is most commonly caused by *S. aureus*, followed by pseudomonas species [18,19]. If an abscess develops, drainage and thorough debridement are necessary. Excision of the medial end of the clavicle, first rib, and manubrium may be required, which usually leaves a large chest wall defect, and exposes major vessels. This defect can be repaired with an advancement or rotational flap of the pectoralis major muscle [20].

Paraspinal muscle infection, a pyogenic infection of skeletal muscle, is rarely reported. Modes of infection include transcutaneous infection by needles or catheters, surgery, blunt trauma, and hematogenous spread from distant sites.

Spondylodiscitis, a term encompassing vertebral osteomyelitis, spondylitis, and discitis, is a rare medical emergency. Spinal epidural abscess is also uncommon, and requires early detection and appropriate treatment to prevent severe morbidity and mortality [21-24]. About 5% of patients with spinal epidural abscesses die, usually because of uncontrolled sepsis, meningitis, or other underlying illnesses [22]. In 25-50% of cases, spondylodiscitis is associated with an epidural abscess or granulation tissue. Pyogenic spondylodiscitis with an epidural abscess may progress to a severe neurological deficit, especially if the diagnosis is established late and it is complicated by the development of an intramedullary abscess of the spinal cord [21].

Hematogenous osteomyelitis usually occurs in patients over 50 years of age and accounts for 3-5% of all cases of osteomyelitis. The incidence of hematogenous osteomyelitis is estimated to be 4 to 24 per million per year in developed countries. Pathogens may infect the spine via three routes: hematogenous spread, direct external inoculation, and spread from contiguous tissues [25]. Spontaneous pyogenic spondylodiscitis usually spreads hematogenously from infections of the skin, subcutaneous tissues, or urinary tract [5]. The hematogenous arterial route predominates, allowing seeding of infection from distant sites to the vertebral column [25]. The most common causative pathogen is *Staphylococcus aureus*, followed in frequency by *BrucellaSalmonella*, and *Mycobacterium tuberculosis*[21,26]. The most likely portal of entry in cases of *S. aureus* infection is the skin (particularly from the skin creases between the toes). Unlike urinary tract infections caused by other pathogens, those caused by *S. aureus* are most often due to hematogenous dissemination. The presence of *S. aureus* in the urine, as in this case, therefore suggests hematogenous spread of infection [27]. *S. aureusStreptococcus* species, and *N. gonorrhoeae* have a high degree of selectivity for the synovium, probably related to adherence characteristics and toxin production [9].

In adults, the vertebral intraosseous metaphyseal artery is an end-artery, and a septic embolism in a metaphyseal artery causes a large wedge-shaped infarct of a subdiscal area of bone. The subsequent spread of infection to the neighboring disc and vertebra creates the characteristic lesion of spondylodiscitis [25]. Ventrally located epidural abscesses in cases such as ours are usually associated with spondylitis and/or discitis [28]. Inoculation is most commonly iatrogenic following spinal surgery, lumbar puncture, or epidural procedures, accounting for 25-30% of cases in some spondylodiscitis series [25]. Mylona et al. described other sources of infection including the genitourinary tract (17%), skin and soft tissue (11%), intravascular devices (5%), gastrointestinal tract (5%), respiratory tract (2%), and the oral cavity (2%) [29]. They found that 12% of patients with pyogenic vertebral osteomyelitis also had infective endocarditis.

## Conclusion

We present a patient with poorly controlled diabetes who developed *S. aureus* septic arthritis of the SCJ following spondylodiscitis. The most likely portal of entry in cases of *S. aureus* septic arthritis is the skin. We identified a recent epidural block as a potential iatrogenic source of infection in our case. It is noteworthy that diabetic patients with *S. aureus* bacteremia may be at risk of severe extended musculoskeletal infections.

## Consent

Written informed consent was obtained from the patient for publication of this case report and any accompanying images. A copy of the written consent is available for review by the Editor-in-Chief of this journal.

## Competing interests

The authors declare that they have no competing interests.

## Authors’ contributions

NS treated the patient and wrote the case report. YI, SK, TM, NM, GT, HM, and YY were involved in the treatment of the patient. SE supervised the writing of this paper and made some major changes after reviewing the first versions. All authors read and approved the final manuscript.

## Pre-publication history

The pre-publication history for this paper can be accessed here:

http://www.biomedcentral.com/1471-227X/12/7/prepub
